# Supplementary figures and images for: Reflecting on motivations: How reasons to publish affect research behaviour in astronomy
Source: PLoS One. 2023 Apr 6;18(4):e0281613. doi: 10.1371/journal.pone.0281613 (PMC10079119; doi:10.1371/journal.pone.0281613)

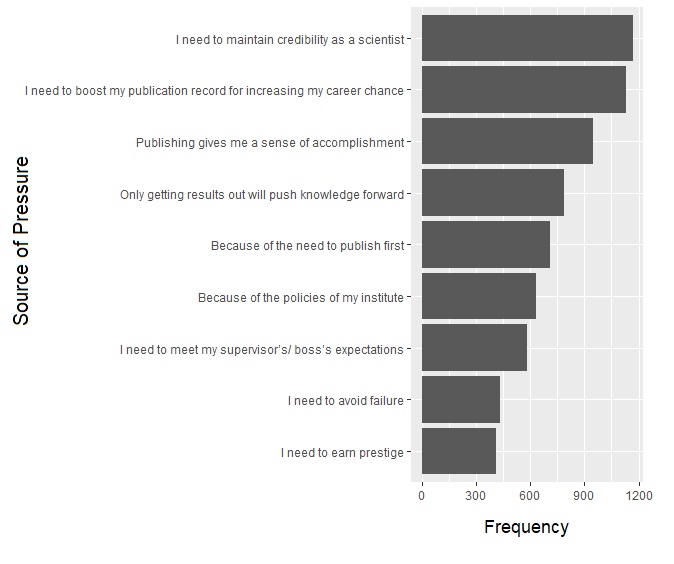

Supplement: S1 Fig — (JPG) [file pone.0281613.s001.jpg]

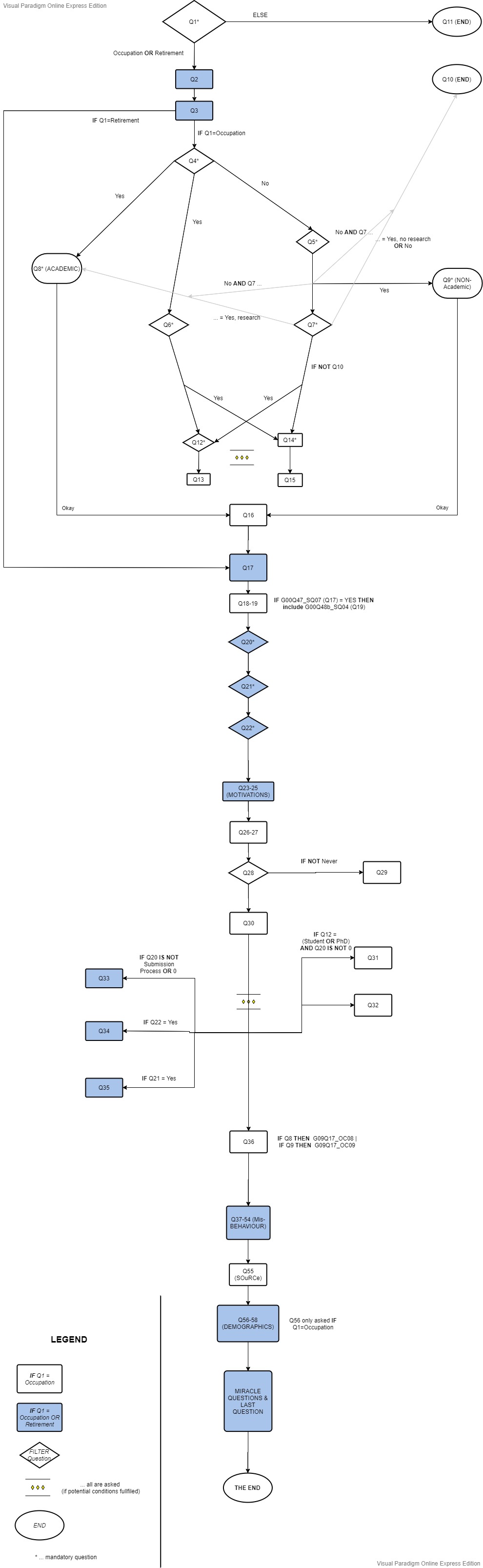

Supplement: S1 File — (ZIP) [file pone.0281613.s005.zip › Heuritsch_Survey2021_Flowchart.jpg]
