# Supplementary material for: Reflecting on motivations: How reasons to publish affect research behaviour in astronomy
Source: PLoS One. 2023 Apr 6;18(4):e0281613. doi: 10.1371/journal.pone.0281613 (PMC10079119; doi:10.1371/journal.pone.0281613)
Supplement: S1 Appendix — (DOCX) [file pone.0281613.s002.docx]

**S1-Appendix: Survey Questions**

**S1-TableS1: Item-battery for the IV M1: “Motivation to become an Astronomer”.**

Question:

“How important were the following motivational factors for you to become an astronomer?”

Items:

Items were designed by the author, and are inspired by Gagné et al. (2015).

For each question the response scale ranged from 1=Strongly Disagree to 5=Strongly Agree.

| Out of curiosity |
| --- |
| I needed a job |
| My goal is to find out more about the laws that govern the universe |
| I enjoy the process of gaining insight in astronomical phenomena |
| Astronomy is a prestigious field in science |
| Being a scientist is a prestigious job |
| I like the intellectual challenge |
| I find basic research more gratifying than the sometimes more profit-oriented activities in other natural sciences |

**S1-TableS2a: Item-battery for the IV M2: “Drivers to Publish”.**

Question:

“What are your personal drivers to publish papers?”

Items:

Items were designed by the author, and are inspired by Gagné et al. (2015).

For each question the response scale ranged from 1=Strongly Disagree to 5=Strongly Agree.

| Publishing is important to share results with the community |
| --- |
| I feel ashamed if I don’t publish |
| Publishing is a requirement from my job |
| Publishing enhances my career prospects |
| I enjoy the review process |
| Publishing my results makes me proud of myself |
| Writing results down has personal significance to me |
| Publishing increases my reputation as a scientist |
| I enjoy the process of writing a paper |

**S1-TableS2b: Item-battery for the IV M3: “Feelings one experiences when not publishing the amount of papers that one aimed to publish”.**

Question:

“How do you feel when you don’t publish the amount of papers that you aimed to publish?”

Items:

Items were designed by the author, and are inspired by Gagné et al. (2015).

For each question the response scale ranged from 1=Strongly Disagree to 5=Strongly Agree and included the option “NA”.

| I feel ashamed |
| --- |
| I feel like I am not a good researcher |
| I feel like I am not doing a good job |
| I feel worthless |
| I am worried that it will negatively impact my career prospects |
| That’s the risk of research that sometimes you are stuck, so I don’t feel any negative emotions |
| I feel disappointed that I cannot share any new insights with my community |
| I am worried that it will negatively impact my research track record |
| I am worried that it will decrease my chances for receiving external grants |
| I am worried that it will decrease my chances for receiving telescope time |

**S1-TableS3: Item-battery for the instrument “Source of the Perceived Publication Pressure”.**

Question:

“What is the source of that pressure? I feel publication pressure, because …”

Items:

Items were designed by the author and were only asked if the survey respondent answered more than “never” in a previous question about “How often do you feel pressure to publish?” (answer options: “never”, “very rarely”, “rarely”, “regularly”, “often”, “very often”).

This was a multiple choice question, where each item could be selected or not selected.

| ... I need to meet my supervisor’s/ boss’s expectations |
| --- |
| ... I need to boost my publication record for increasing my career chance |
| ... publishing gives me a sense of accomplishment |
| ... I need to earn prestige |
| ... I need to avoid failure |
| ... of the need to publish first |
| ... only getting results out will push knowledge forward |
| ... I need to maintain credibility as a scientist |
| ... of the policies of my institute |

**S1-TableS4: Item-battery for the instrument “Most rewarding aspects about work”.**

Question:

“What do you find most rewarding about your work?”

Items:

Items were designed by the author.

This was a ranking question, where a survey respondent chose 3 answers in a ranked order.

| Enjoying the process of finding truths about the universe |
| --- |
| Receiving praise from a colleague/ my supervisor |
| Making incremental steps in building up knowledge |
| Making ground-breaking steps in building up knowledge |
| Winning scientific prizes |
| Receiving a job promotion (a more senior job title) |
| Receiving a salary raise |
| Getting a paper published |
