# Supplementary material for: Reflecting on motivations: How reasons to publish affect research behaviour in astronomy
Source: PLoS One. 2023 Apr 6;18(4):e0281613. doi: 10.1371/journal.pone.0281613 (PMC10079119; doi:10.1371/journal.pone.0281613)
Supplement: S2 Appendix — (DOCX) [file pone.0281613.s003.docx]

**S2-Appendix: EFAs & CFAs**

**S2-TableS1a: M1 – CATPCA (4 factors)**

This table displays the SPSS output of the 4-factor CATPCA of the M1 construct consisting of 8 items (see *Table S1 in S1*). N=1244 since the EFA is performed on half of the data set (~50% randomly chosen) and the CFA (*S2-TableS4*) on the other half.

| **Pattern Matrix** | | | | |
| --- | --- | --- | --- | --- |
| M1 Items | **Dimension** | | | |
|  | Introjected Regulation | Identified Regulation | Intrinsic Motivation | Residual Category |
| Out of curiosity | -.224 | .402 | .161 | .554 |
| I needed a job | .172 | -.225 | -.105 | .861 |
| My goal is to find out more about the laws that govern the universe | .098 | .666 | .101 | -.085 |
| I enjoy the process of gaining insight in astronomical phenomena | .018 | -.029 | .956 | -.039 |
| Astronomy is a prestigious field in science | .913 | .059 | .063 | .013 |
| Being a scientist is a prestigious job | .883 | .086 | .002 | .083 |
| I like the intellectual challenge | .045 | -.059 | .964 | -.015 |
| I find basic research more gratifying than the sometimes more profit-oriented activities in other natural sciences | .084 | .871 | -.174 | -.074 |

**S2-TableS1b: M1 – Types of Motivation; Theory versus CATPCA**

Comparison between the categorisation as expected from theory and as obtained from the CATPCA for the types of motivation of the M1 construct. Differing results are denoted by a *.

| **M1 Items** | **Type of Motivation (as expected from theory)** | **Type of Motivation (results from CATPCA;** *from TableS1a in S2***)** |
| --- | --- | --- |
| Out of curiosity | Intrinsic Motivation | Residual Category* |
| I needed a job | External Regulation | Residual Category* |
| My goal is to find out more about the laws that govern the universe | Identified Regulation | Identified Regulation |
| I enjoy the process of gaining insight in astronomical phenomena | Intrinsic Motivation | Intrinsic Motivation |
| Astronomy is a prestigious field in science | Introjected Regulation | Introjected Regulation |
| Being a scientist is a prestigious job | Introjected Regulation | Introjected Regulation |
| I like the intellectual challenge | Intrinsic Motivation | Intrinsic Motivation |
| I find basic research more gratifying than the sometimes more profit-oriented activities in other natural sciences | Identified Regulation | Identified Regulation |

**S2-TableS1c: M1 – Cronbach Alphas before removal of items**

This table displays the SPSS output of the Cronbach alphas of the M1 construct consisting of 8 items. * denotes items that I subsequently removed.

| **Reliability Statistics** | |
| --- | --- |
| Cronbachs Alpha | N of Items |
| .608 | 8 |

| **Item-Total-Statistics** | | | | |
| --- | --- | --- | --- | --- |
| M1 Items | Scale Mean if Item Deleted | Scale Variance if Item Deleted | Corrected Item-Total Correlation | Cronbach’s Alpha if Item Deleted |
| Out of curiosity* | 25.49 | 17.388 | .221 | .601 |
| I needed a job* | 27.22 | 18.102 | .072 | .658 |
| My goal is to find out more about the laws that govern the universe | 25.42 | 17.019 | .331 | .569 |
| I enjoy the process of gaining insight in astronomical phenomena | 25.06 | 17.472 | .388 | .562 |
| Astronomy is a prestigious field in science | 26.43 | 15.019 | .421 | .537 |
| Being a scientist is a prestigious job | 26.58 | 15.089 | .433 | .533 |
| I like the intellectual challenge | 25.11 | 17.310 | .419 | .556 |
| I find basic research more gratifying than the sometimes more profit-oriented activities in other natural sciences | 25.64 | 16.668 | .306 | .576 |

**S2-TableS1d: cleaned M1 – Cronbach Alphas after removal of items**

This table displays the SPSS output of the Cronbach alphas of the cleaned M1 construct after removal of items. which resulted in 6 remaining items.

| **Reliability Statistics** | |
| --- | --- |
| Cronbachs Alpha | N of Items |
| .666 | 6 |

| **Item-Total-Statistics** | | | | |
| --- | --- | --- | --- | --- |
| Cleaned M1 Items | Scale Mean if Item Deleted | Scale Variance if Item Deleted | Corrected Item-Total Correlation | Cronbach’s Alpha if Item Deleted |
| My goal is to find out more about the laws that govern the universe | 19.00 | 11.554 | .351 | .639 |
| I enjoy the process of gaining insight in astronomical phenomena | 18.64 | 11.883 | .430 | .620 |
| Astronomy is a prestigious field in science | 20.01 | 9.743 | .454 | .603 |
| Being a scientist is a prestigious job | 20.16 | 10.027 | .434 | .611 |
| I like the intellectual challenge | 18.70 | 11.927 | .427 | .621 |
| I find basic research more gratifying than the sometimes more profit-oriented activities in other natural sciences? | 19.22 | 11.165 | .333 | .648 |

**S2-TableS1e: cleaned M1 – CATPCA (2 factors)**

This table displays the SPSS output of the 2-factor CATPCA of the cleaned M1 construct after removal of items. which resulted in 6 remaining items. N=1245 since the EFA is performed on half of the data set (~50% randomly chosen) and the CFA (*S2-TableS4*) on the other half.

| **Pattern Matrix** | | |
| --- | --- | --- |
| Cleaned M1 Items | **Dimension** | |
|  | M1F1- Autonomous Motivation | M1F2 - Controlled Motivation |
| My goal is to find out more about the laws that govern the universe | .599 | .067 |
| I enjoy the process of gaining insight in astronomical phenomena | .914 | -.075 |
| Astronomy is a prestigious field in science | .046 | .915 |
| Being a scientist is a prestigious job | -.001 | .916 |
| I like the intellectual challenge | .895 | -.051 |
| I find basic research more gratifying than the sometimes more profit-oriented activities in other natural sciences? | .527 | .154 |

**S2-TableS2a: M2 – CATPCA (4 factors)**

This table displays the SPSS output of the 4-factor CATPCA of the M2 construct consisting of 9 items (see *Table S2a in S1*). N=1088 since the EFA is performed on half of the data set (~50% randomly chosen) and the CFA (*S2-TableS4*) on the other half.

| **Pattern Matrix** | | | | |
| --- | --- | --- | --- | --- |
| M2 Items | **Dimension** | | | |
|  | External Regulation | Introjected Regulation | Identified Regulation | Intrinsic Motivation |
| Publishing is important to share results with the community | .058 | .000 | .933 | -,042 |
| I feel ashamed if I don’t publish | .451 | .319 | -.220 | -,077 |
| Publishing is a requirement from my job | .958 | -.276 | .136 | ,090 |
| Publishing enhances my career prospects | .598 | .370 | -.002 | -,028 |
| I enjoy the review process | .179 | -.067 | -.254 | ,890 |
| Publishing my results makes me proud of myself | -.162 | .897 | -.034 | ,104 |
| Writing results down has personal significance to me | -.046 | .159 | .303 | ,577 |
| Publishing increases my reputation as a scientist | .318 | .604 | .131 | -,060 |
| I enjoy the process of writing a paper | -.107 | .065 | .072 | ,813 |

**S2-TableS2b: M2 – Types of Motivation; Theory versus CATPCA**

Comparison between the categorisation as expected from theory and as obtained from the CATPCA for the types of motivation of the M2 construct. Differing results are denoted by a *.

| **M2 Items** | **Type of Motivation (as expected from theory)** | **Type of Motivation (results from CATPCA;** *from TableS2a in S2***)** |
| --- | --- | --- |
| Publishing is important to share results with the community | Identified Regulation | Identified Regulation |
| I feel ashamed if I don’t publish | Introjected Regulation | External Regulation* |
| Publishing is a requirement from my job | External Regulation | External Regulation |
| Publishing enhances my career prospects | External Regulation | External Regulation |
| I enjoy the review process | Intrinsic Motivation | Intrinsic Motivation |
| Publishing my results makes me proud of myself | Introjected Regulation | Introjected Regulation |
| Writing results down has personal significance to me | Identified Regulation | Intrinsic Motivation* |
| Publishing increases my reputation as a scientist | Introjected Regulation | Introjected Regulation |
| I enjoy the process of writing a paper | Intrinsic Motivation | Intrinsic Motivation |

**S2-TableS2c: M2 – Cronbach Alphas**

This table displays the SPSS output of the Cronbach alphas of the M2 construct consisting of 9 items.

| **Reliability Statistics** | |
| --- | --- |
| Cronbachs Alpha | N of Items |
| .662 | 9 |

| **Item-Total-Statistics** | | | | |
| --- | --- | --- | --- | --- |
| M2 Items | Scale Mean if Item Deleted | Scale Variance if Item Deleted | Corrected Item-Total Correlation | Cronbach’s Alpha if Item Deleted |
| Publishing is important to share results with the community | 28.01 | 22.699 | .275 | .648 |
| I feel ashamed if I don’t publish | 29.33 | 21.292 | .208 | .669 |
| Publishing is a requirement from my job | 28.55 | 21.199 | .272 | .650 |
| Publishing enhances my career prospects | 28.59 | 20.241 | .411 | .618 |
| I enjoy the review process | 30.07 | 21.224 | .307 | .641 |
| Publishing my results makes me proud of myself | 28.68 | 19.891 | .466 | .606 |
| Writing results down has personal significance to me | 28.98 | 20.316 | .385 | .624 |
| Publishing increases my reputation as a scientist | 28.53 | 20.311 | .471 | .608 |
| I enjoy the process of writing a paper | 29.32 | 20.735 | .301 | .644 |

**S2-TableS2d: M2 – CATPCA (2 factors)**

This table displays the SPSS output of the 2-factor CATPCA of the M2 construct consisting of 9 items. N=1008 since the EFA is performed on half of the data set (~50% randomly chosen) and the CFA (*S2-TableS4*) on the other half.

| **Pattern Matrix** | | |
| --- | --- | --- |
| M2 Items | **Dimension** | |
|  | M2F1- Autonomous Motivation | M2F2 - Controlled Motivation |
| Publishing is important to share results with the community | .509 | .035 |
| I feel ashamed if I don’t publish | -.142 | .600 |
| Publishing is a requirement from my job | -.140 | .696 |
| Publishing enhances my career prospects | -.009 | .808 |
| I enjoy the review process | .691 | -.066 |
| Publishing my results makes me proud of myself | .457 | .434 |
| Writing results down has personal significance to me | .740 | .015 |
| Publishing increases my reputation as a scientist | .153 | .723 |
| I enjoy the process of writing a paper | .837 | -.175 |

**S2-TableS3a: M3 – CATPCA (3 factors)**

This table displays the SPSS output of the 3-factor CATPCA of the M3 construct consisting of 10 items (see *Table S2b in S1*). N=660 since the EFA is performed on half of the data set (~50% randomly chosen) and the CFA (*S2-TableS4*) on the other half.

| **Pattern Matrix** | | | |
| --- | --- | --- | --- |
| M3 Items | **Dimension** | | |
|  | External Regulation | Introjected Regulation | Residual Category |
| I feel ashamed | -.043 | .907 | .110 |
| I feel like I am not a good researcher | -.046 | .910 | .048 |
| I feel like I am not doing a good job | -.027 | .898 | .084 |
| I feel worthless | -.029 | .887 | .069 |
| I am worried that it will negatively impact my career prospects | .806 | .129 | -.092 |
| That’s the risk of research that sometimes you are stuck. so I don’t feel any negative emotions | -.125 | -.551 | .431 |
| I feel disappointed that I cannot share any new insights with my community | .123 | .191 | .926 |
| I am worried that it will negatively impact my research track record | .805 | .145 | -.110 |
| I am worried that it will decrease my chances for receiving external grants | .908 | -.126 | .100 |
| I am worried that it will decrease my chances for receiving telescope time | .734 | -.162 | .214 |

**S2-TableS3b: M3 – Types of Motivation; Theory versus CATPCA**

Comparison between the categorisation as expected from theory and as obtained from the CATPCA for the types of motivation of the M3 construct.

There is no difference between the categorisation based on theory and the factors of the CATPCA (see *TableS3a in S2*). The residual category of the CATPCA consists of the single item representing identified regulation and the control item (in italics). Pearson correlations of all items with the control item were as expected negative and this item was subsequently removed from further analysis.

| **M3 Items** | **Type of Motivation** |
| --- | --- |
| I feel ashamed | Introjected Regulation |
| I feel like I am not a good researcher | Introjected Regulation |
| I feel like I am not doing a good job | Introjected Regulation |
| I feel worthless | Introjected Regulation |
| I am worried that it will negatively impact my career prospects | External Regulation |
| That’s the risk of research that sometimes you are stuck. so I don’t feel any negative emotions | Residual Category (*Control Item*) |
| I feel disappointed that I cannot share any new insights with my community | Residual Category (*Identified Regulation*) |
| I am worried that it will negatively impact my research track record | External Regulation |
| I am worried that it will decrease my chances for receiving external grants | External Regulation |
| I am worried that it will decrease my chances for receiving telescope time | External Regulation |

**S2-TableS3c: cleaned M3 – Cronbach Alphas**

This table displays the SPSS output of the Cronbach alphas of the cleaned M3 construct consisting of 9 items.

| **Reliability Statistics** | | | | | | |
| --- | --- | --- | --- | --- | --- | --- |
| Cronbachs Alpha | N of Items |  |  |  |  |  |
| .867 | 9 |  |  |  |  |  |
| **Item-Total-Statistics** | | | | | | |
| Cleaned M3 Items | | | Scale Mean if Item Deleted | Scale Variance if Item Deleted | Corrected Item-Total Correlation | Cronbach’s Alpha if Item Deleted |
| I feel ashamed | | | 26.00 | 50.513 | .699 | .843 |
| I feel like I am not a good researcher | | | 25.60 | 50.909 | .695 | .844 |
| I feel like I am not doing a good job | | | 25.36 | 52.143 | .690 | .845 |
| I feel worthless | | | 26.23 | 51.374 | .666 | .847 |
| I am worried that it will negatively impact my career prospects | | | 25.08 | 51.541 | .687 | .845 |
| I feel disappointed that I cannot share any new insights with my community | | | 25.43 | 59.764 | .304 | .877 |
| I am worried that it will negatively impact my research track record | | | 25.00 | 52.660 | .693 | .845 |
| I am worried that it will decrease my chances for receiving external grants | | | 24.74 | 54.988 | .548 | .858 |
| I am worried that it will decrease my chances for receiving telescope time | | | 25.41 | 57.164 | .408 | .870 |

**S2-TableS3d: cleaned M3 – CATPCA (3 factors)**

This table displays the SPSS output of the 3-factor CATPCA of the cleaned M3 construct consisting of 9 items. N=674 since the EFA is performed on half of the data set (~50% randomly chosen) and the CFA (*S2-TableS4*) on the other half.

| **Pattern Matrix** | | | |
| --- | --- | --- | --- |
| Cleaned M3 Items | **Dimension** | | |
|  | M3F3- External Regulation | M3F2- Introjected Regulation | M3F1- Identified Regulation |
| I feel ashamed | -,024 | ,896 | ,084 |
| I feel like I am not a good researcher | -,023 | ,919 | -,031 |
| I feel like I am not doing a good job | -,003 | ,896 | ,014 |
| I feel worthless | -,007 | ,891 | ,006 |
| I am worried that it will negatively impact my career prospects | ,818 | ,143 | -,094 |
| I feel disappointed that I cannot share any new insights with my community | ,049 | ,056 | 1,025 |
| I am worried that it will negatively impact my research track record | ,818 | ,152 | -,111 |
| I am worried that it will decrease my chances for receiving external grants | ,892 | -,112 | ,075 |
| I am worried that it will decrease my chances for receiving telescope time | ,709 | -,150 | ,155 |

**S2-TableS4: Results of the comparative factor analyses (CFAs) the motivation constructs M1-M3.**

N=1752 since the CFA is performed on half of the data set (~50% randomly chosen) and the EFAs (*see former tables in this Appendix*) on the other half.

Note: These results take significant covariations between indicators into account.

| **Independent Variable** | **Comparative Fit Index (CFI)** | **Tucker-Lewis Index (TLI)** | **Root Mean Square Error of Approximation (RMSEA)** |
| --- | --- | --- | --- |
| M1 | 0.992 | 0.983 | 0.037 90%CI(.017, .057). |
| M2 | 0.928 | 0.856 | 0.081 90%CI(.069, .094). |
| M3 | 0.970 | 0.954 | 0.077 90%CI(.063, .091). |
