# Supplementary material for: Reflecting on motivations: How reasons to publish affect research behaviour in astronomy
Source: PLoS One. 2023 Apr 6;18(4):e0281613. doi: 10.1371/journal.pone.0281613 (PMC10079119; doi:10.1371/journal.pone.0281613)
Supplement: S3 Appendix — (DOCX) [file pone.0281613.s004.docx]

**S3-Appendix: Results**

**S3-TableS1: Descriptive Statistics of M1.**

This table displays the SPSS output of the descriptive statistics of the M1 construct consisting of 8 items (see *Table S1 in S1*) and its factors (see *Table S1e in S2*).

|  |  |  |  |  |
| --- | --- | --- | --- | --- |
| Factor | Item | N | Mean | Sdt.- Deviation |
| *Removed* | Out of curiosity | 2496 | 4.09 | 1.124 |
| *Removed* | I needed a job | 2494 | 2.30 | 1.303 |
| M1F1 | My goal is to find out more about the laws that govern the universe | 2501 | 4.17 | .992 |
| M1F1 | I enjoy the process of gaining insight in astronomical phenomena | 2507 | 4.55 | .757 |
| M1F2 | Astronomy is a prestigious field in science | 2502 | 3.12 | 1.268 |
| M1F2 | Being a scientist is a prestigious job | 2496 | 2.98 | 1.236 |
| M1F1 | I like the intellectual challenge | 2502 | 4.48 | .765 |
| M1F1 | I find basic research more gratifying than the sometimes more profit-oriented activities in other natural sciences | 2501 | 3.97 | 1.109 |

The descriptive statistics of the items that make up factors M1F1 and M1F2 yield that the comparatively biggest driver to become an astronomer is the “enjoyment of the process of gaining insight in astronomical phenomena” (mean=4.55), followed by “liking the intellectual challenge” (mean=4.48) and “finding out more about the laws that govern the universe” (mean=4.17), while the smallest driver is “needing a job” (mean=2.3).

**S3-TableS2: Descriptive Statistics of M2.**

This table displays the SPSS output of the descriptive statistics of the M2 construct consisting of 9 items (see *Table S2a in S1*) and its factors (see *Table S2d in S2*).

| Factor | Item | N | Mean | Sdt.- Deviation |
| --- | --- | --- | --- | --- |
| M2F1 | Publishing is important to share results with the community | 2035 | 4.51 | .746 |
| M2F2 | I feel ashamed if I don’t publish | 2030 | 3.16 | 1.269 |
| M2F2 | Publishing is a requirement from my job | 2034 | 3.97 | 1.109 |
| M2F2 | Publishing enhances my career prospects | 2032 | 3.94 | 1.065 |
| M2F1 | I enjoy the review process | 2032 | 2.44 | 1.081 |
| M2F1 | Publishing my results makes me proud of myself | 2033 | 3.81 | 1.069 |
| M2F1 | Writing results down has personal significance to me | 2030 | 3.54 | 1.103 |
| M2F2 | Publishing increases my reputation as a scientist | 2034 | 3.97 | .974 |
| M2F1 | I enjoy the process of writing a paper | 2034 | 3.22 | 1.175 |

The descriptive statistics of the items that make up factors M2F1 and M2F2 yield that the comparatively biggest driver to publish is an autonomous one, “publishing is important to share results with the community” (mean=4.51), closely followed by “publishing is a requirement from my job” (mean=3.97) and “publishing increases my reputation as a scientist” (mean=3.97).

**S3-TableS3: Descriptive Statistics of M3.**

This table displays the SPSS output of the descriptive statistics of the M3 construct consisting of 10 items (see *Table S2b in S1*) and its factors (see *Table S3d in S2*).

| Factor | Item | N | Mean | Sdt.- Deviation |
| --- | --- | --- | --- | --- |
| M3F2 | I feel ashamed | 1893 | 2.59 | 1.397 |
| M3F2 | I feel like I am not a good researcher | 1921 | 3.01 | 1.388 |
| M3F2 | I feel like I am not doing a good job | 1924 | 3.26 | 1.306 |
| M3F2 | I feel worthless | 1884 | 2.38 | 1.368 |
| M3F3 | I am worried that it will negatively impact my career prospects | 1890 | 3.63 | 1.308 |
| *removed* | That’s the risk of research that sometimes you are stuck, so I don’t feel any negative emotions | 1881 | 2.78 | 1.203 |
| M3F1 | I feel disappointed that I cannot share any new insights with my community | 1919 | 3.19 | 1.168 |
| M3F3 | I am worried that it will negatively impact my research track record | 1910 | 3.63 | 1.223 |
| M3F3 | I am worried that it will decrease my chances for receiving external grants | 1836 | 3.91 | 1.187 |
| M3F3 | I am worried that it will decrease my chances for receiving telescope time | 1510 | 3.26 | 1.271 |

Similarly to the analysis of the M2 construct (*S3-TableS2*), respondents’ comparatively biggest worries when not publishing the amount of papers that they aimed to publish concern a possible negative impact on their career: “I am worried that it will decrease my chances for receiving external grants” (mean=3.91), “I am worried that it will negatively impact my career prospects” (mean=3.63) and “I am worried that it will negatively impact my research track record” (mean=3.63).

**S3-TableS4: Ranking of what astronomers find most rewarding about their work.**

| **Rank** | **Item** | **n** |
| --- | --- | --- |
| 1 | Enjoying the process of finding truths about the universe | 1934 |
| 2 | Making incremental steps in building up knowledge | 1582 |
| 3 | Making ground-breaking steps in building up knowledge | 1398 |
| 4 | Getting a paper published | 1268 |
| 5 | Receiving praise from a colleague/ my supervisor | 609 |
| 6 | Receiving a job promotion (a more senior job title) | 316 |
| 7 | Receiving a salary raise | 207 |
| 8 | Winning scientific prizes | 182 |

2500 astronomers responded to the ranking question “What do you find most rewarding about your work?”. Most astronomers chose “enjoying the process of finding truths about the universe” (n=1076) for rank 1. N=652 chose “making incremental steps in building up knowledge” as rank 2 and n=702 selected “getting a paper published” as rank 3. When summing up all responses for all three ranks, the ranking presented in this table was obtained, were “making ground-breaking steps in building up knowledge” took the 3^rd^ place before “getting a paper published”. The least important rewards, both, in the individual rank answers and when summed up were chosen as “receiving a job promotion”, “winning scientific prizes” and “receiving a salary raise”.

**S3-FigureS1: Descriptive Statistics of the additional instruments.**

1951 astronomers responded to the multiple choice question what the source of their perceived publication pressure is. Results are visualised in this figure. The two most chosen answers are “I need to maintain credibility as a scientist” (N=1167) and “I need to boost my publication record for increasing my career chance” (N=1128). The least chosen source of publication pressure were “I need to earn prestige” (N=412) and “I need to avoid failure” (N=436).

**FigureS1.** Source of perceived publication pressure, ranked by frequency of the respondents’ answers.

**S3-TableS5: Regression models of DV = M1F1 (N=1360) & M1F2 (N=1357); regressed onto the control variables. * indicates statistical significance (p < 0.05).**

|  | **M1F1:  Autonomous Motivation to become an astronomer** | | | | | **M1F2:  Controlled Motivation to become an astronomer** | | | | |
| --- | --- | --- | --- | --- | --- | --- | --- | --- | --- | --- |
|  | **Unstandardized Coefficients** | | **Stand. Coeff.** | **t** | **Sig.** | **Unstandardized Coefficients** | | **Stand. Coeff.** | **t** | **Sig.** |
|  | **B** | **Std. Error** | **Beta** |  |  | **B** | **Std. Error** | **Beta** |  |  |
| Intercept: | 4.287 | 0.098 |  | 43.914 | <0.001 * | 3.083 | 0.178 |  | 17.343 | <0.001 * |
| Gender: Male | -0.084 | 0.039 | -0.061 | -2.183 | 0.029 * | 0.157 | 0.070 | 0.062 | 2.239 | 0.025 * |
| Position: PhD Candidate | -0.098 | 0.069 | -0.053 | -1.415 | 0.157 | -0.092 | 0.126 | -0.028 | -0.736 | 0.462 |
| Position: Postdoc | -0.090 | 0.050 | -0.061 | -1.799 | 0.072 | -0.243 | 0.092 | -0.090 | -2.657 | 0.008 * |
| Position: Assistant Prof. | -0.089 | 0.068 | -0.039 | -1.306 | 0.192 | -0.191 | 0.124 | -0.046 | -1.545 | 0.123 |
| Position: Associate Prof. | -0.041 | 0.056 | -0.023 | -0.731 | 0.465 | -.085 | 0.102 | -0.026 | -0.833 | 0.405 |
| Position: Other | -0.105 | 0.059 | -0.057 | -1.779 | 0.076 | -0.255 | 0.108 | -0.076 | -2.367 | 0.018 * |
| Primary Employer: Academic | 0.059 | 0.074 | 0.023 | 0.800 | 0.424 | 0.237 | 0.134 | 0.050 | 1.770 | 0.077 |
| Papers published: Submission | -0.039 | 0.122 | -0.010 | -0.319 | 0.750 | -.094 | 0.222 | -0.013 | -0.422 | 0.673 |
| Papers published: 0 | -0.075 | 0.098 | -0.022 | -0.763 | 0.446 | 0.116 | 0.178 | 0.019 | 0.653 | 0.514 |
| Papers published: 6–20 | 0.047 | 0.046 | 0.035 | 1.025 | 0.305 | -0.124 | 0.083 | -0.050 | -1.496 | 0.135 |
| Papers published: >20 | 0.121 | 0.047 | 0.090 | 2.552 | 0.011 * | -0.080 | 0.086 | -0.033 | -.925 | 0.355 |
| Location: Global North | 0.021 | 0.047 | 0.012 | 0.449 | 0.653 | -0.203 | 0.085 | -0.065 | -2.382 | 0.017 * |

This table presents the regression models for the autonomous & controlled motivation to become an astronomer in dependence of the control variables. Male as opposed to female/ non-binary tend to feel less autonomous motivation to become an astronomer (by 0.084 points). Having published more than 20 papers in the last 5 years is related to a higher autonomous motivation to become an astronomer (by 0.121 points). The controlled motivation to become an astronomer by contrast, tends to be higher for males as compared to females/ non-binaries (by 0.157 points). Postdocs and astronomers from the position category “Other” perceive less controlled motivation to become an astronomer than Full Professors (by 0.243 & 0.255 points, respectively). Astronomers employed by an institution in the Global North also report less controlled motivation to become an astronomer as compared to the Global South (by 0.203 points).
