# Supplementary material for: Reflecting on motivations: How reasons to publish affect research behaviour in astronomy
Source: PLoS One. 2023 Apr 6;18(4):e0281613. doi: 10.1371/journal.pone.0281613 (PMC10079119; doi:10.1371/journal.pone.0281613)
Supplement: Attachment — Submitted filename: Review PLOS.docx [file pone.0281613.s006.docx]

The article describes a large survey conducted among astronomers to understand how their motivations play a role in publishing and research behaviour. The topic is timely and the insights are useful, but although PLOS ONE does not dictate word limits, I think the piece would be more useful when written up more concisely. I have a few suggestions that I hope to be useful for the author in revising the piece:

**General remarks**

It may help the author to think a bit more about how the various hypotheses help answer the main research question, and to pay more attention to those (i.e., research questions) in the Introduction, Results and Discussion section. This may mean that substantial number of Tables should be moved to the supplementary information, so that the information is available to interested readers, but that general readers get a bit more support from the author in piecing together the different models.

I also wanted to note that the author says all data is available, but I saw no link to a public repository or a raw dataset anywhere. This may be due to the type of consent participants have provided, but then the author should explain this.

**Abstract**

Most of the abstract is devoted to the Introduction, and it lacks crucial details on the methodology employed and the key results. It is inappropriate to write “in summary, we find…” when no results have been presented beforehand. Please revise by briefly summarising the main research leading up to this work (1-2 sentences), followed by a description of its methodology, including a description of the participants, response rate, measures employed, etc, followed by key results.

**Introduction**

It is unclear from the Introduction (and it remains unclear throughout the manuscript) how the results relate to previous work by the author (in particular, Heuritsch 2021b). It seems to me that the preprint cited, and the current manuscript describe the same study, but that the current manuscript *also* includes items related to motivational factors. If that is true, this may help substantially shortening the manuscript and it would be fine, provided the author explicitly acknowledged the fact that this and the cited study above rely on mostly the same data.

What is missing is an explanation as to why the study focuses on astronomers, and whether the author expects their theoretical framework to work as described for astronomers, or for scientists in general.

This may be a matter of style, but the section on how the paper will be structured on p.4 can be left out in my view, it does not add much.

It seems to me that the various theories that the author touches upon already relate to each other in some way or another. It might be helpful to start with Fig. 1 and then explain the different theoretical predictions that flow from it and relate those to the research questions.

It struck me as somewhat odd that the research questions only appeared in the middle of the Methods section, it would be helpful to have them appear at the end of the Introduction, connected with the literature reviewed before, and connected with the various hypotheses. This last part, connecting the research questions with the hypotheses might also be helpful for the reader to better follow the authors’ line of reasoning.

**Methods**

In relation to the above comment on whether the same survey underlies the Heuritsch 2021b report and the current manuscript, if instruments have already been explained in a detail, a brief summary would be sufficient.

Given the large amount of independent and dependent variables, it might be useful to have them in a table instead.

It also seems to me that to answer the research questions, it would be most helpful to focus on the multivariate regression models where the different dependent variables are presented together (also because they might also relate). This could perhaps be helpful way of shortening the variety of models to be reviewed in the main manuscript and move some of the separate analyses on one dependent variable only to the supplementary information.

**Results**

Perhaps I’m mistaken, but it seems that the EFA and CFA have been run on the same dataset which I think is not correct. I am not a statistician, but I believe this practice is generally not recommended (see also: https://us.sagepub.com/sites/default/files/upm-assets/106745_book_item_106745.pdf)

For Table 1 and 2 (and for the remainder of the tables), no need to repeat the descriptions in the Table in the text preceding and following the Table.

It could be helpful to connect the different analysis explicitly to the Hypotheses and Research Questions again, as now the reader must go back and forth quite a bit to make sense of the order in which the Results are presented, and to understand how they interrelate.

When comparing the different motivation and control models, it might be useful to display them all in a big table so the reader can easily see which regression coefficients changed depending on the DV used. Similarly, it might be more informative to see turnover intentions for academics and non-academics side-by-side.

I am wondering, for the sake of not overwhelming the reader if section 4.5 is not better placed in a supplement. The author could simply consider writing that they also tried to refine previously published models to see if it influenced their results and summarise this, without displaying all the models (I realise this might lead to leaving out parts of the research question/some hypotheses), but I think it may help the reader grasping the overall message.

Minor remark: no need to repeat the reference categories in the Discussion (p.19-20).

The Results section contains hints of interpretations, whereas these interpretations are better placed in the Discussion (e.g., p. 30 “This may be partly due the difference in N…”).

**Discussion**

At the risk of sounding repetitive, I would like to see the research questions feature more strongly in the discussion, and to read the author answer those (if needed be partially based on the different hypotheses).

I also found the Discussion repeating the Results quite a bit, but perhaps by putting the Results in a smaller set of larger tables and limiting the text around these tables can already help prevent this.

It seems that the references featured in the Discussion were largely the same as in the Introduction, making me miss some contextualisation here. Are these results unique for the particular subgroup studied? What sort of new questions do they raise?

As for limitations, I miss the consideration of a potential response bias. What could this mean for the author’s interpretations?

I wish the author much success in revising the description of this study!
