# Supplementary material for: Reflecting on motivations: How reasons to publish affect research behaviour in astronomy
Source: PLoS One. 2023 Apr 6;18(4):e0281613. doi: 10.1371/journal.pone.0281613 (PMC10079119; doi:10.1371/journal.pone.0281613)
Supplement: Attachment — Submitted filename: Response to Reviewers.docx [file pone.0281613.s007.docx]

This reply letter contains: First, general remarks to both reviewers. Second, a reply to the editor. Third, a reply to Reviewer 1 and fourth, a reply to Reviewer 2.

**GENERAL REMARKS TO BOTH REVIEWERS:**

Dear both reviewers,

First of all, thank you for your review and for your generally constructive, straightforward and useful feedback, which I feel has helped me a lot in improving the paper!

Let me respond to some topics here, which both of you remarked, and I will reply to individual comments below.

1. Especially your suggestion to connect the hypotheses better with the research questions and to guide the reader along the paper by featuring these more, has – in my opinion – made the paper indeed a lot clearer. I decided to not put as many parts into supplementary material as you may have suggested (although I did remove some parts and shortened the page count by 9%), because I feel like structuring the paper more along the hypotheses helps the reader to have a better overview of that “swimming pool of data” (Reviewer 2) and he/ she may decide to skip a section (e.g. 4.5.) if they are not interested in that particular hypothesis.
2. As for your remarks that I should make the connection to Heuritsch (2021a; now [12]) clearer, I was surprised since I really thought I did that (yes, both studies are based on the same web-bases survey, however, analyse of course overlapping or different instruments). However, if you felt that this connection wasn’t clear enough, I absolutely agree that it needed to be explicitly clarified! I hope that the way I improved this leaves no question about the connection open. However, if you have any suggestion, how I could still make the relationship between the two studies clearer, please do let me know!
3. As for the abstract, both of you also had about the same remarks, so I tried to adapt it now according to your suggestions.
4. As for the limitations: Most of those strengths/limitations you mentioned are actually described in the previous paper (2021a; now [12]). Reviewer 1 pointed out that there is no need (provided that I made the connection to this paper clearer) to repeat that kind of information. I agree and that is why I chose to refer to the complete analysis of the limitations in Heuritsch (2021a; now [12]) at the beginning of the limitations. I hope this is sufficient.

**RESPONSE TO EDITOR:**

Dear editor,

Thank you for handling the peer review and here you find responses to the additional requirements you mentioned in your email (your text in green):

Journal Requirements:

When submitting your revision, we need you to address these additional requirements.

1. Please ensure that your manuscript meets PLOS ONE's style requirements, including those for file naming. The PLOS ONE style templates can be found at

<https://journals.plos.org/plosone/s/file?id=wjVg/PLOSOne_formatting_sample_main_body.pdf>  and

<https://journals.plos.org/plosone/s/file?id=ba62/PLOSOne_formatting_sample_title_authors_affiliations.pdf>

🡪 Done! I am sorry – I must have used an old template (I am pretty sure I followed one before submission).

2. Please provide additional details regarding participant consent. In the ethics statement in the Methods and online submission information, please ensure that you have specified what type you obtained (for instance, written or verbal, and if verbal, how it was documented and witnessed). If your study included minors, state whether you obtained consent from parents or guardians. If the need for consent was waived by the ethics committee, please include this information.

🡪 Done (in the Methods section)

3. For this single-authored manuscript, please replace "we" with "I".

🡪 Done!

*Please change "female” or "male" to "woman” or "man" as appropriate, when used as a noun (see for instance <https://apastyle.apa.org/style-grammar-guidelines/bias-free-language/gender>).

🡪 This would not an appropriate change in this case, since the answer categories gave the option to the participants to choose between „Female, „Male“, „Non-Binary“ and „Would rather not disclose“.

**RESPONSE TO REVIEWER 1:**

Dear Reviewer 1,

Please find the responses to your comments here (your text in green):

The article describes a large survey conducted among astronomers to understand how their motivations play a role in publishing and research behaviour. The topic is timely and the insights are useful, but although PLOS ONE does not dictate word limits, I think the piece would be more useful when written up more concisely. I have a few suggestions that I hope to be useful for the author in revising the piece:

**General remarks**

It may help the author to think a bit more about how the various hypotheses help answer the main research question, and to pay more attention to those (i.e., research questions) in the Introduction, Results and Discussion section. This may mean that substantial number of Tables should be moved to the supplementary information, so that the information is available to interested readers, but that general readers get a bit more support from the author in piecing together the different models.

- Replied to in general remarks to both reviewers.

I also wanted to note that the author says all data is available, but I saw no link to a public repository or a raw dataset anywhere. This may be due to the type of consent participants have provided, but then the author should explain this.

- Yes, you are absolutely right. The reason why I hadn’t submitted it with the last manuscript is, because I have been in discussion with my supervisor about the best format and platform for the publication of the data of the whole survey. We intend to fully share all data that my PhD thesis is based on (and the survey is part of it). This process has, unfortunately, taken longer than expected. You may be pleased to hear, however, that I am now sharing the data relevant for this particular paper in S0 File.

**Abstract**

Most of the abstract is devoted to the Introduction, and it lacks crucial details on the methodology employed and the key results. It is inappropriate to write “in summary, we find…” when no results have been presented beforehand. Please revise by briefly summarising the main research leading up to this work (1-2 sentences), followed by a description of its methodology, including a description of the participants, response rate, measures employed, etc, followed by key results.

**Introduction**

It is unclear from the Introduction (and it remains unclear throughout the manuscript) how the results relate to previous work by the author (in particular, Heuritsch 2021b). It seems to me that the preprint cited, and the current manuscript describe the same study, but that the current manuscript *also* includes items related to motivational factors. If that is true, this may help substantially shortening the manuscript and it would be fine, provided the author explicitly acknowledged the fact that this and the cited study above rely on mostly the same data.

- Yes, your perception is correct! I conducted a large survey (it took 30min to 1hr to fill in). In 2021a [now 12] I analysed a part of this data set (mostly how publication pressure and perception of justice affects perceived misbehaviour). With this paper, I bring motivational factors into play. I have tried to make the connection with 2021a more clear by adding a sentence in line 56. In 2021b [now 14], I studied astronomer’s motivational factors qualitatively and how those relate to what counts in the field (institutional norms) and rewards. Hence, 2021a is quantitative, drawing on OCT; 2021b is qualitative, drawing on RCT and this study now brings both together. I have tried to make this more clear in the next now.

What is missing is an explanation as to why the study focuses on astronomers, and whether the author expects their theoretical framework to work as described for astronomers, or for scientists in general.

- Since this study is a follow up on Heuritsch (2021a [now 12]) and Heuritsch (2021b [now 14]) where I explained why I chose astronomy as a field to study from a meta perspective, I failed doing this in this paper again. However, yes, you are right that I work from the assumption that science study findings on astronomy may be generalizable to academia in general. I now added a few sentences explaining all of this to the Introduction (line 45 to 54).

This may be a matter of style, but the section on how the paper will be structured on p.4 can be left out in my view, it does not add much.

- I am okay with removing this section, however, Reviewer 2 praised this (“Nice to see how the author elaborates on how the paper is structured!”). I would like to ask the editor what to do …

It seems to me that the various theories that the author touches upon already relate to each other in some way or another. It might be helpful to start with Fig. 1 and then explain the different theoretical predictions that flow from it and relate those to the research questions.

It struck me as somewhat odd that the research questions only appeared in the middle of the Methods section, it would be helpful to have them appear at the end of the Introduction, connected with the literature reviewed before, and connected with the various hypotheses. This last part, connecting the research questions with the hypotheses might also be helpful for the reader to better follow the authors’ line of reasoning.

- Thank you for this suggestion! I think it’s indeed better to have the RQs and Hypotheses at the end of the introduction! So I revised them now, connected the RQs with the Hs and added them as section 2.3 instead of previously 3.3.

**Methods**

In relation to the above comment on whether the same survey underlies the Heuritsch 2021b report and the current manuscript, if instruments have already been explained in a detail, a brief summary would be sufficient.

- Please note that it is Heuritsch 2021a (not b) which describes this survey. And yes, indeed I only give a brief summary of the instruments that were used therein and I refer to where they are described in detail (example: “The instrument is described in detail elsewhere (Heuritsch, 2021a: p.7) […]”).

Given the large amount of independent and dependent variables, it might be useful to have them in a table instead.

- I have followed the format of presenting IVs and DVs of Martinson et al. (2006), which I found most appropriate, since many instruments used in this study (and Heuritsch 2021a) are based on this study (and other referenced related ones) and this paper serves as a contribution to those studies. However, if the editor finds a table more suitable, I will change the format.

It also seems to me that to answer the research questions, it would be most helpful to focus on the multivariate regression models where the different dependent variables are presented together (also because they might also relate). This could perhaps be helpful way of shortening the variety of models to be reviewed in the main manuscript and move some of the separate analyses on one dependent variable only to the supplementary information.

- Yes, thank you, I have now done this with the tables were your comment was applicable.

**Results**

Perhaps I’m mistaken, but it seems that the EFA and CFA have been run on the same dataset which I think is not correct. I am not a statistician, but I believe this practice is generally not recommended (see also: https://us.sagepub.com/sites/default/files/upm-assets/106745_book_item_106745.pdf)

- Thank you for spotting this – I corrected for it now (in S2-Appendix) and in the Manuscript. Unsurprisingly, this didn’t lead to significant changes in the results of the CFAs and EFAs, so that the subsequent analyses are still accurate.

For Table 1 and 2 (and for the remainder of the tables), no need to repeat the descriptions in the Table in the text preceding and following the Table.

- I don’t know exactly what you mean. If you mean that I describe in the text what the regression analyses yields – this is how the statistics papers that my study is based on do it. It seems common practice to report on the results around the tables and then discuss (interprete) the results in the Discussion section (as you remark in one of your further comments).

It could be helpful to connect the different analysis explicitly to the Hypotheses and Research Questions again, as now the reader must go back and forth quite a bit to make sense of the order in which the Results are presented, and to understand how they interrelate.

- Yes, absolutely – good idea! I have now related the result sections with the hypotheses and hope that the relationships between the different analyses have become clearer to the reader now.

When comparing the different motivation and control models, it might be useful to display them all in a big table so the reader can easily see which regression coefficients changed depending on the DV used. Similarly, it might be more informative to see turnover intentions for academics and non-academics side-by-side.

- Yes! Also did with all tables where applicable like with your related comment above.

I am wondering, for the sake of not overwhelming the reader if section 4.5 is not better placed in a supplement. The author could simply consider writing that they also tried to refine previously published models to see if it influenced their results and summarise this, without displaying all the models (I realise this might lead to leaving out parts of the research question/some hypotheses), but I think it may help the reader grasping the overall message.

- No – Integrating the motivational factors into the model from my earlier study (2021a) was the main purpose of this work ☺. The purpose was to explore relationships between motivation, publication pressure, perceptions of justice and finally observing misbehaviour. I hope that I could make this clearer with several adaptations in the text now.

Minor remark: no need to repeat the reference categories in the Discussion (p.19-20).

- Thank you – I took it out now!

The Results section contains hints of interpretations, whereas these interpretations are better placed in the Discussion (e.g., p. 30 “This may be partly due the difference in N…”).

- Thank you, I tried to move these where applicable.

**Discussion**

At the risk of sounding repetitive, I would like to see the research questions feature more strongly in the discussion, and to read the author answer those (if needed be partially based on the different hypotheses).

- Yes, absolutely! I added answers to the RQs now to the Discussion section

I also found the Discussion repeating the Results quite a bit, but perhaps by putting the Results in a smaller set of larger tables and limiting the text around these tables can already help prevent this.

- As I remarked earlier, this seems to be common practice in the statistics papers that my study is based on; The results report on what the regression tables yield (the relevant information at least) and in the Discussion section interpretes the results (so what this means for the hypotheses, research questions and how this relates to previous research, etc). I hope however, that the changes I have made in the text also make this section more straight forward.

It seems that the references featured in the Discussion were largely the same as in the Introduction, making me miss some contextualisation here. Are these results unique for the particular subgroup studied? What sort of new questions do they raise?

- This study is put into the *context* of the studies mentioned in the Introduction, which is why in the Discussion section I discuss how my results fit in there (or not). As for the two questions you raised here, I tried to answer this now by adding a few sentences in the Discussion. The paragraph on why I chose astronomy that I added in the Introduction may also help answering your question regarding the subgroup.

As for limitations, I miss the consideration of a potential response bias. What could this mean for the author’s interpretations?

- Replied to in general remarks to both reviewers.

I wish the author much success in revising the description of this study!

- Thank you! ☺

**RESPONSE TO REVIEWER 2:**

Dear Reviewer 2,

Please find the responses to your comments here (your text in green):

Thank you for having the opportunity to read and review with manuscript which I did with joy and pleasure. Great work and great way to further dive into the motivations of researchers. A couple of suggestions belolw that may improve the manuscript:

**Abstract:**
Please assure that others also know what is meant with reflexive metrics. I know the author is very experienced with the term but PLOS One readers may not and should be taken by hand :)

- I added a clarification here.

Please make sure that readers know what anomie is

- I deleted anomie from the abstract, as that may be too much information at this point anyway.

Now the abstract does not contain any methods and I think this should be captures in more detail in the abstract. What is done? What Questionnaires are used, what types of participants, what hypothesis etc. I think the IMRaD format can help here.

- Replied to in general remarks to both reviewers.

**Introduction:**
How is resaerch culture shaping resaerch behaviour. Is there evidence for this?
And how is what types of behaviour shaped by what elements of culture?

- There is a whole strand of research, which studies the relation between research culture and research behavior. In the introduction I gave a few examples as of how research culture shapes research behavior (line 36-42). This was maybe not that clear so I added “For example,” now in line 36.

Is 10% of the variance a reference to the work of Heuritsch 2021?

- Yes, I made that more clear now.

Maybe elaborate on what the author means with institutional norms? They sound vague and not so concrete. More specific? What is considered these norms?

- I gave an example for an institutional norm in astronomy (publishing a specific amount of paper per year) in line 77-78. Heuritsch (2021b [now 14]) elaborates on what exactly the institutional norms are in astronomy, but giving more examples would go to far here. However, I added a definition for what institutional norms are now in line 69-70.

It is a bit weird to talk about we, while it is a single author paper.

- I have changed this now to “I” or adapted otherwise appropriately.

Nice to see how the author elaborates on how the paper is structured!

Are there any RQs worth mentioning in the introduction? IOW: why this paper? What is the urgency?

- Replied to in general remarks to both reviewers.

**Methods:**
Matter of style, but response rate etc is oftentimes reported in the results section.
How is the estimation of 13000-15000 calculated. It sounds a bit too far off and it is a bit weird to calculate a response rate, while you dont know how many people are reasched. Better to hightlihgt this limitation in discussion section, which you most likely do.

- I have now added information about this in line 261-262.

**Instruments:**
Are the 18 items in the supplementary material? How are these 18 items chosen and why?
Make sure that all the instruments are supplemented in these sections.

- The 18 items of scientific (mis-)behavior – and some other instruments, where indicated – are described in Heuritsch (2021a [now 12]). I have now made the connection of this study and 2021a clearer, so I hope that you also find (like Reviewer 1) that a summary and reference to 2021a is sufficient as opposed to describing all instruments in detail again.

What was the result fo the factor analysis of the M1 instrument? Please report this in the paper

- This is actually reported in section 4.1.

Motivation to publish: what did you do now and why? the paragraph is a bit unclear. Is there any psychometric validation? What constructs and concepts are measured?

- I believe M2 is described clearly in the respective paragraph. I have added a sentence now to make M3 clearer also (line 347-349). The paragraph explains also that the concepts measured are the various forms of motivations (external regulation, introjected regulation, identified regulation and intrinsic motivation) one could have to publish papers. The instrument is described in detail in the Supporting Information.

What are the ethical considerations and assessment by an ERB?
Is there a privacy policy?
Is there informed consent?

- I have added this information to the Methods section now.

Is there a preregistration with these hypothesis somewhere available?

- No, this was not required for this study or in my field.

**Results:**
I feel that I am a bit lost in all the tables and details. What does the author want to say? What are the key findings? What is the main focus of the paper? Is there a content that should be focused on.
I suggest to highlight the most important data in 3 tables and move the rest to the supplementary materials

- As I described earlier, I have now tried to structure the results more along the hypotheses for the reader to follow the analyses easier. I have also reduced the numbers of tables. However, maybe not to the extent you would have hoped for ;). I realise this was a big study, and I hope my adaptations make it easier for the reader now to decide which hypotheses they are interested in and read the respective analyses only.

**Discussion:**

The limitations section is very short to put it mildly. What are the strengths of the paper?
and what are the limitations (response rate, response bias, confirmation bias? Reporting bias? No valid Qs? no generalisation? Comparison with other countries about levels?

- Replied to in general remarks to both reviewers.

What is your main conclusion after all these data that you have collected? What are the main questionnaires that are used that give the most valuable information? Now it feels a bit as swimming in a swimming pool with too many data

- I hope to have made this clearer now by adding a short paragraph with the two main conclusions. I also hope that structuring the paper along the hypotheses and RQs now has given a better overview over the swimming pool ;).
